# Correction: Retraction: Robust policy evaluation from large-scale observational studies

**DOI:** 10.1371/journal.pone.0355824

**Published:** 2026-08-13

**Authors:** The PLOS One Editors

## Notice of Republication

The retraction notice [1] for [2] was republished on July 28, 2026, to correct an error in the References list. The publisher apologizes for the error.

Please download the retraction notice again to view the correct version. The originally published, uncorrected notice and the republished, corrected notice are provided here for reference.

## Supporting information

S1 File Originally published, uncorrected retraction notice. (PDF)

S2 File Republished, corrected retraction notice. (PDF)
