# Supplementary material for: Correction: Retraction: Robust policy evaluation from large-scale observational studies
Source: PLoS One. 2026 Aug 13;21(8):e0355824. doi: 10.1371/journal.pone.0355824 (PMC13472359; doi:10.1371/journal.pone.0355824)
Supplement: Supplementary file 2 [file pone.0355824.s002.pdf]

PLOS One

RETRACTION

Retraction: Robust policy evaluation from large-scale observational studies

The *PLOS One* Editors

After publication of this article [1], the corresponding author notified *PLOS One* of an error in the p-value calculation caused by a coding error, affecting Fig 4 and related text in the Experiment and result subsection of [1].

Following input from a member of the Editorial Board, the *PLOS One* Editors concluded that the conclusion drawn from applying the mathematical model to the large-scale validation dataset, is incorrect. Specifically, after correcting the error, the revised analysis shows no causal relation between the Medicare Hospital Readmission Reduction Program (HRRP) and non-index readmissions. However, the evaluation of the model's scalability remains sound, and the study's methodological contribution is unaffected.

As the manuscript discusses the causal relationship extensively, and this forms a central aim of the study, the *PLOS One* Editors consider that a key conclusion is no longer upheld. To address this concern, the *PLOS One* Editors retract this article and have published an updated version of the article containing the corrected p-value calculation, an updated Fig 4, and revised conclusions [2].

All authors agreed with the retraction.

References

1. Islam MS, Morshed MS, Young GJ, Noor-E-Alam M. RETRACTED: Robust policy evaluation from large-scale observational studies. PLoS One. 2019;14(10):e0223360. https://doi.org/10.1371/journal.pone.0223360 PMID: 31603910
2. Islam MS, Morshed MS, Young GJ, Noor-E-Alam M. Robust policy evaluation from large-scale observational studies. PLoS One. 2026;21(6):e0348228. https://doi.org/10.1371/journal.pone.0348228

Check for updates

OPEN ACCESS

Citation: The *PLOS One* Editors (2026) Retraction: Robust policy evaluation from large-scale observational studies. PLoS One 21(6): e0352001. https://doi.org/10.1371/journal.pone.0352001

Published: June 30, 2026

Copyright: © 2026 The *PLOS One* Editors. This is an open access article distributed under the terms of the Creative Commons Attribution License, which permits unrestricted use, distribution, and reproduction in any medium, provided the original author and source are credited.
